# Receiving screened donor human milk as part of a community‐based lactation support programme reduces parental symptoms of anxiety and depression

**DOI:** 10.1111/mcn.13686

**Published:** 2024-06-19

**Authors:** Amy Brown, Sam Griffin, Gillian Weaver, Natalie Shenker

**Affiliations:** ^1^ Centre for Lactation, Infant Feeding and Translation (LIFT) Swansea UK; ^2^ Faculty of Medicine, Health and Life Sciences Swansea University Swansea UK; ^3^ The Human Milk Foundation Victory Road Berkhamsted UK; ^4^ Department of Surgery and Cancer Imperial College London London UK

**Keywords:** anxiety, breastfeeding, depression, donor human milk, infant feeding, lactation support, mental health

## Abstract

Infant feeding decisions and maternal mental health are closely tied. Donor human milk (DHM) protects premature infant health and development and can reduce hospital stays. Recent qualitative research has highlighted that having the option for an infant to receive DHM can also support parental wellbeing through reducing concerns about infant health and supporting feeding preferences. However, no quantitative study has examined this relationship. In this study, anxiety and depression scores were measured before and after receiving DHM using the Hospital Anxiety and Depression Scale for 80 parents (77 mothers, 3 fathers) who had sought DHM from a community‐facing milk bank. Reasons for seeking DHM included maternal cancer, maternal and infant health complications, insufficient glandular tissue, and low milk supply. Open‐ended questions explored the experience of receiving milk. Milk bank records were used to match details of milk given (volume, duration, exclusivity, lactation support given) with survey responses. Both anxiety and depression scores significantly reduced after receiving milk. Although greater lactation support and longer duration of milk predicted a greater decrease in scores, in a regression analysis, only volume of milk given remained a significant predictor. Almost all parents agreed that being able to access DHM supported their wellbeing predominantly through reducing anxieties around infant health but also through feeding choices being respected and the support given at difficult times. The findings add important considerations to the literature considering when and for whom DHM should be used and the complex interplay between infant feeding and mental health.

## Key messages

- Receiving donor human milk (DHM) alongside lactation support from a community‐facing milk bank was associated with a significant decrease in anxiety and depression scores in parents seeking DHM due to maternal cancer, maternal or infant health reasons, or low supply.
- A higher volume of milk, seeking milk for maternal health reasons and more lactation support were associated with reductions in anxiety or depression scores. However, when considered together, only the volume of milk remained significant.
- Parents attributed reduced concerns for infant health, recognition of infant feeding preferences and the care and support given as reducing anxiety and depression symptoms.

## Graphical abstract

Receiving donor human milk (DHM) alongside lactation support from a community milk bank was associated with a decrease in anxiety and depression scores in parents seeking DHM for maternal or infant health reasons, or low supply. Parents attributed this to reduced health concerns, respect of feeding choices and support received.

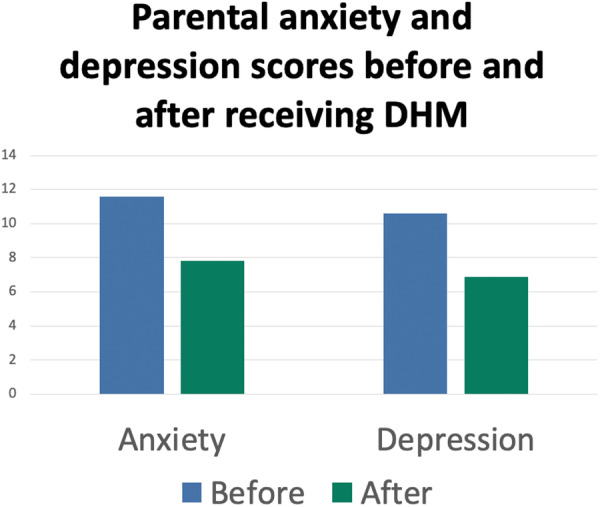

## 1 INTRODUCTION

The complex link between breastfeeding and maternal mental health is well‐established (Alimi et al., 2022; Brown, 2018; Da Silva Tanganhito et al., 2020). For mothers who *want* to breastfeed, meeting their personal breastfeeding goals can support wellbeing and increase parenting satisfaction (Avilla et al., 2020; Shepherd et al., 2017). This can be particularly impactful when an infant is unwell or has been born prematurely (Flacking et al., 2016; Hookway et al., 2023). Conversely, experiencing breastfeeding difficulties or stopping if women are not ready to do so can be associated with feelings of guilt, failure and frustration (Thomson et al., 2015; Jackson et al., 2021). It can increase risk of post‐natal depression (Borra et al., 2015), impact upon perceived bonding (Roth et al., 2021) and leave women with feelings of lasting guilt and grief at not being able to mother and care for their infant in the way that they had intended (Brown, 2019).

The relationship between breastfeeding and maternal mental health is in part driven by increased maternal anxieties around infant health and development (Odom et al., 2013) and the impact of pain and exhaustion (Huang et al., 2023; Kendall‐Tackett, 2007). However, the relationship is more complex than this. Breastfeeding goals are often dismissed as being inconsequential because formula milk is available, but infant feeding preferences are not simply about ensuring a baby is fed. Breastfeeding intentions may be driven by cultural, religious or environmental reasons, meaning that stopping has further social consequences (Hohl et al., 2016; Joffe et al., 2019; Mehrpisheh et al., 2020). The decision to stop breastfeeding can be tied up in health complications, challenging circumstances such as an early return to work, a lack of support from family and health professionals, and feelings of inconvenience and expense (Dutheil et al., 2021; Feenstra et al., 2018; Hvatum & Glavin, 2017).

One aspect that is often not considered within discussions around infant feeding and mental health is where donor human milk (DHM) from a human milk bank fits into the discourse. Most of the research examining the impact of DHM has focussed on infant health and developmental outcomes. Typically, DHM is offered in neonatal care units to infants under a gestation of less than 32 weeks or weighing less than 1500 g when the mother's own milk (MOM) is unavailable or insufficient to meet their baby's requirements (Shenker et al., 2023). DHM can reduce the risk of necrotising enterocolitis and complications such as bronchopulmonary dysplasia in premature infants (Quigley et al., 2019; Villamor‐Martínez et al., 2018). When offered alongside optimal lactation support, it may also help mothers to increase their own supply in the early days following a premature birth, acting as a bridge to full breastfeeding (Brown & Shenker, 2022; Merjaneh et al., 2020; Williams et al., 2016).

However, recent new guidelines published by the British Association of Perinatal Medicine emphasised that parental wishes regarding feeding should be considered and recognise that DHM can help to support maternal wellbeing (BAPM, 2023). Qualitative studies have highlighted that DHM can support maternal wellbeing and reduce anxiety in the neonatal unit (Cassidy & Dykes, 2019) or for late pre‐term and term babies who were receiving supplemental DHM (Kair & Flaherman, 2017). A qualitative survey with 107 parents in the United Kingdom who had received DHM for their baby on the neonatal unit or through a charity due to maternal health issues such as cancer, also found parents felt that receiving DHM‐supported their wellbeing. DHM reduced anxiety over infant health, but also the experience of receiving DHM supported wellbeing due to parents feeling listened to and respected, and that their infant feeding preferences were valued (Brown & Shenker, 2022).

These data point to a potential protective effect of receiving DHM upon parental mental health. Limitations of the current evidence include a focus on retrospective design, no quantitative studies, and a lack of clinical measures of anxiety and depression. The aim of the current study was, therefore, to measure symptoms of anxiety and depression in parents before and after their infant received DHM.

## 2 METHODS

### 2.1 Setting

Parents who contacted the Hearts Milk Bank in the United Kingdom to enquire about using DHM for their babies participated in this study. In the United Kingdom, DHM is typically mainly available on neonatal units and often reserved for premature infants born at <32 weeks gestation. However, Hearts Milk Bank has a limited supply of DHM for use within community settings, according to set referral criteria (Griffin et al., 2022). Recipients of this milk include mothers who cannot breastfeed due to contraindicated medical treatment (such as chemotherapy and contraindicated medications), previous mastectomy or older infants who are significantly unwell when a mother has stopped breastfeeding. Other recipients include breastfeeding mothers who either have a permanently reduced milk supply due to hypoplasia or who are working on building their own supply.

In general, 4 weeks of DHM is provided where breastfeeding is not possible, and support may continue for several weeks or even months when maternal supply can be established. Families can request DHM directly from the milk bank, accompanied by a request from a clinician involved in the care of their infant (e.g., paediatrician, general practitioner, International Board Certified Lactation Consultant [IBCLC]). Support with using DHM, alongside lactation support for mothers building their own supply or partially breastfeeding, is provided to all families by an IBCLC employed by the milk bank, with assistance from a team of breastfeeding counsellors and midwives. This is provided via phone and email as the milk bank is based in a research and enterprise park rather than a health care facility and is often some distance from the parents' home. Milk is taken to them by Blood Bikers or a member of the wider team.

### 2.2 Participants

All parents who contacted the milk bank between January 2020 and April 2021 and met inclusion criteria were sent an invitation to also participate in the research. Inclusion criteria were parents aged 16+, able to complete the survey in the English language and able to give informed consent. The decision was made to allow inclusion of those aged 16–17 because they were parents describing their care and are considered by the NHS Health Research Authority to be capable of giving informed consent. It was made clear to parents that participation would be anonymous, unknown to the milk bank, and would not affect their experience of receiving milk or any support provided.

Participation was limited to one parent, but either parent could take part. We included fathers/partners in the study because they are often instrumental in organising and collecting DHM and maybe the main point of contact. They may also be sourcing milk for an adopted infant or be caring solely for their infant if the mother is critically unwell or has died.

All participants gave informed consent before completing the survey. The questionnaire contained a study information sheet and consent questions. Participants read through full study information and inclusion criteria before consenting to take part in the proposed research by ticking agreement. Only when all consent items were agreed did the full questionnaire load. Participants could also contact the milk bank or the research team if they had any further questions before taking part.

### 2.3 Measures

Participants completed two questionnaires via an online survey link hosted via Qualtrics. The first questionnaire was completed after they had made their request for DHM but before they received the milk. It included:

- Demographic details (parental sex, age, education, location).
- Donor milk recipient identification number (allocated by the milk bank) to allow for matching of pre‐ and post‐questionnaires.
- Details of the infant/s to receive DHM for (age/week of pregnancy, sex).
- Reason for seeking DHM, for example, maternal cancer, low supply.
- Current milk feeding status of their infant, if relevant (breastfeeding duration and exclusivity, formula use, DHM use).
- A copy of the Hospital Anxiety and Depression Scale (HADS) (Zigmond & Snaith, 1983).

The HADS questionnaire measures symptoms of anxiety and depression over the last 7 days. It contains seven items for each sub‐scale, which are scored in terms of how frequently an individual experiences that emotion (i.e., feeling cheerful or restless). Possible scores range from 0 to 21 for each sub‐scale. Scores are then grouped into ‘Normal’ (0–7), ‘Borderline abnormal’ (8–10) and ‘Abnormal’ (11–21) separately for both anxiety and depression.

The HADS is one of the most used tools to measure anxiety and depression in research and is considered to have strong internal consistency (Bjelland et al., 2002). The tool was initially developed to screen for mental health difficulties among those with physical illness and, as a result, does not include somatic symptoms, that is, insomnia, loss of appetite or fatigue (Zigmond & Snaith, 1983). This makes it a useful tool to measure post‐natal wellbeing as new parents are commonly affected by insomnia and fatigue, independently to any mental health issue. It has also been used in several studies exploring infant feeding and maternal mental health (e.g., Arifunhera et al., 2016; Fukui et al., 2021).

The second questionnaire was sent to participants who received DHM. It was sent to participants after the last support contact, on completion of the use of DHM. It contained:

- Donor milk recipient Identification number;
- A further copy of the HADS;
- Feeding method post‐receiving donor milk (breast, formula, any other maternal milk);
- Open‐ended text boxes exploring experience of receiving DHM and perceived impact upon wellbeing (Table 1). These questions were designed to be broad, open‐ended questions with prompts aimed at helping establish whether any changes to mental health and wellbeing were linked to receiving milk rather than time simply passing or symptoms easing.

**Table 1:** Open‐ended survey questions exploring experiences of receiving donor human milk.

What was your experience of receiving donor milk? For example, how did it make you feel or what difference did it have to you, your baby or your family?
How did your experience of donor milk fit with your expectations of receiving donor milk? Was it better? Different? More challenging than expected?
How do you feel about how you are currently feeding your baby? Can you tell us a little more about why you feel that way?
Do you have any further comments on the experience of receiving donor milk?

### 2.4 Procedure

For both questionnaires, participants were sent a link to access the questionnaire online, although a paper copy was available on request if necessary. The invitation to complete the survey was sent by S. G. at Hearts Milk Bank to ensure that participant details were not shared without prior consent with the research team. However, to ensure the confidentiality of participants, only the researchers could see who had participated, for example, the milk bank was not aware of participation during the period where participants were receiving milk from them. Only A. B., who was not part of the milk bank team and therefore did not know recipients, accessed the full data set that included recipient numbers. Once completed, a debrief statement was given, explaining the study, thanking them for participation, and giving them contact details for support organisations if needed.

On completion of data collection, a list of recipient numbers who had completed both surveys was sent to N. S. at Hearts Milk Bank by A. B. N. S. provided details of volume of milk and exclusivity, number of support emails and calls given for each recipient. These were matched to survey responses. Recipient numbers were then removed from sharable copies of the data set and replaced with a new matched participant number from 1 to 80.

### 2.5 Data analysis

Data were analysed using SPSS version 27. Participant pre‐ and post‐questionnaires were matched. Data were only used for matched pairs; those completing only a pre‐ or post‐questionnaire were not included in this analysis. The HADS was scored as per instructions to give two scales of anxiety and depression. Scores were also grouped into normal (0–7), borderline abnormal (8–10) and abnormal (11–21). We also grouped reasons for seeking donor milk into two categories: a milk supply issue (to include low milk supply, surrogacy and infant health) and maternal health (developmental anomaly, maternal cancer, bilateral mastectomy, maternal health issue). These two groupings represented different reasons for needing DHM. The decision to include surrogacy and infant health in the milk supply issue was that both essentially result in a need for milk for the infant, separate from maternal health. It also likely has a different psychological impact, that is, a mother might not face the same expectation or pressure to produce milk for an older infant who is unwell compared to a newborn infant. We included developmental anomaly in the maternal health issue as it represented a physiological health complication that would be unlikely to change even with high‐quality support.

Descriptive statistics were used to compute score frequencies and categories. Paired *t*‐tests were used to compare differences in anxiety and depression scores pre‐ and post‐receiving DHM. Cohen's *d* was used to calculate effect size. Correlations between anxiety and depression scores were computed, followed by a regression analysis for significant predictors. For the open‐ended text boxes, a thematic analysis was conducted using a simple descriptive analysis (Sandelowski, 2010). The first author immersed themselves in the data, reading through responses from each participant and across questions for all participants. Next responses were read and reread to identify smaller themes. These smaller themes were then grouped into larger sub‐themes (Braun & Clarke, 2006). To enhance trustworthiness of the data (Lincoln & Guba, 1986), initial coding was completed by A. B., with N. S. reviewing proposed themes and sub‐themes.

### 2.6 Ethical statement

Ethical approval was granted by the Research Ethics Committee at the College of Human and Health Sciences, Swansea University. All aspects of the study were carried out in line with the declaration of Helsinki.

## 3 RESULTS

Eighty parents (77 mothers and 3 fathers) provided full pre‐ and post‐service data. In total, 116 pre‐questionnaires were sent (completed by 108 parents, 93.1%), and 108 post‐questionnaires. The overall complete response rate (i.e., both pre‐ and post‐completed) was 74.1%. The mean age of participants was 35.36 years (SD: 4.66) with a range from 25 to 47 years. Full demographic data is shown in Table 2.

**Table 2:** Demographic background of parent participants.

Category	Sub‐category	*N*	%
Age	18–24	0	0.0
	25–29	6	7.5
	30–34	32	40.0
	35–39	26	32.5
	40–44	14	17.5
	45+	2	2.5
Education	GCSE or equivalent	1	1.3
	A level or equivalent	6	7.5
	Degree or equivalent	30	37.5
	Post‐graduate qualification or equivalent	40	50.0
	Question not answered	3	3.8
Ethnicity	Asian or Asian British: Chinese	1	1.3
	Asian or Asian British: Indian	2	2.5
	White or White British or Irish	70	87.6
	Mixed or Multiple	2	2.5
	Other	3	3.8
	Question not answered	2	2.5
Employment	Full time	51	63.7
	Part time	13	16.3
	No	14	17.5
	Question not answered	2	2.5
Marital status	Married/civil partnership	52	65.0
	Cohabiting	24	30.0
	Single	1	1.3
	Question not answered	3	3.8

DHM was received for 83 infants (including three sets of twins). Twenty‐five participants (31.3%) made their first contact with the milk bank during pregnancy and 55 (68.8%) after their baby was born. The average age of the baby at the end of receiving DHM was 11.83 weeks (SD: 13.12 weeks) with a range from 1 to 104 weeks. Most babies were younger, with 78/80 babies being under 12 weeks at the end of receiving milk. The two older babies received milk for infant health issues. Half of babies (*n* = 40) received DHM for 8 weeks or less.

Table 3 shows the reasons for seeking DHM alongside the mean volume received and the percentage of cases in each reason group that received exclusive (i.e., full feeds) or partial (i.e., alongside MOM or formula milk) DHM. Overall, 31.3% (*n* = 25) received exclusive DHM feeds and 68.8% (*n* = 55) partial DHM. Those who received exclusive feeds received significantly more DHM than those who received partial feeds [*t* (78) = 5.215, *p* ≤ 0.001]. A mean volume of 29.31 (SD: 11.44) litres was received by those in the exclusive group compared to 14.86 (SD: 11.51) in the partial group. All babies who received exclusive DHM received it due to reasons preventing breastfeeding.

**Table 3:** Reasons for seeking donor milk, volume received and exclusive versus partial.

	Recipients			Exclusive		Partial	
Indication	*N*	%	Mean volume of DHM received (L) (SD)	*N*	%	*N*	%
Low supply	41	51.2	13.7 (11.9)	0	0.0	41	51.2
Developmental anomaly^a^	10	12.5	24.0 (11.9)	3	3.8	7	8.8
Maternal cancer	10	12.5	37.3 (6.13)	9	11.3	1	1.3
Maternal ill health^b^	8	10.0	18.4 (12.1)	5	6.3	3	3.8
Surrogacy	6	7.5	17.0 (7.77)	5	6.3	1	1.3
Bilateral mastectomy	3	3.8	27.6 (5.13)	3	3.8	0	0.0
Infant ill health	2	2.5	20.0 (14.1)	0	0	2	2.5

^a^ Breast anomalies included insufficient glandular tissue, breast hypoplasia and previous surgery for breast hypertrophy.

^b^ Maternal ill health included HIV, need for medication contraindicated for breastfeeding, pituitary tumour.

For those who received partial milk (*n* = 55), 50 babies (90.9%) also received maternal milk. For the three babies who did not, donor milk was requested due to maternal health issues and cancer, preventing breastfeeding. After donor milk provision had stopped, 42 babies (52.5% of the full sample or 84.0% of those who received partial milk) continued to be breastfed. Of these babies who continued to be breastfed, 19 also had some formula (45.2%).

Table 4 shows the mean anxiety and depression scores pre‐ and post‐receiving DHM, alongside the mean reduction in score. Paired sample *t*‐tests found a significant difference between pre‐ and post‐scores for anxiety [*t* (79) = 9.536, *p* < 0.001; Cohen's *d* = 3.57) and depression [*t* (79) = 9.701; Cohen's *d* = 3.35]. The number of fathers in the sample was too small to conduct meaningful statistical comparisons in the score. However, the mean reduction in anxiety and depression scores was similar for both mothers and fathers.

**Table 4:** Mean anxiety and depression scores pre‐ and post‐receiving DHM, alongside change in scores for the full sample, mothers and fathers.

	Pre‐DHM	Post‐DHM	Change full sample	Change mothers (*n* = 77)	Change fathers (*n* = 3)
Anxiety	11.58 (SD: 2.63)	7.77 (SD: 2.58)	−3.81 (SD: 3.57)	−3.79 (SD: 3.64)	−4.33 (SD: 0.57)
Depression	10.60 (SD: 1.97)	6.96 (SD: 2.61)	−3.64 (SD: 3.35	−3.70 (SD: 3.32)	−3.33 (SD: 3.21)

Abbreviation: DHM, donor human milk.

Table 5 shows the categorisation of pre‐ and post‐scores into ‘normal’, ‘borderline abnormal’ and ‘abnormal’. Before receiving DHM, over 90% of parents were classed as having borderline or abnormal anxiety and depression scores. Post‐receiving DHM, large increases were made in the number of parents considered to have ‘normal’ levels of anxiety and depression from pre‐ to post‐scores, for example, before receiving DHM, 7.5% of the sample had ‘normal’ levels of anxiety, increasing to 48.8% post‐receiving milk. Likewise, for depression, 3.8% had normal levels of anxiety before receiving milk, increasing to 57.5% post‐receiving milk. A Wilcoxon signed ranks test found this change to be significant for anxiety (*Z* = −6.058, *p* < 0.01) and depression (*Z* = 6.284, *p* < 0.001).

**Table 5:** Categorisation of anxiety and depression scores pre‐ and post‐receiving donor milk.

	Normal		Borderline abnormal		Abnormal	
	*N*	%	*N*	%	*N*	%
Pre‐anxiety	6	7.5	20	25.0	54	67.5
Post‐anxiety	39	48.8	27	33.8	14	17.5
Pre‐depression	3	3.8	35	43.8	42	52.5
Post‐depression	46	57.5	26	32.5	8	10.0

Pearson's correlations were used to explore the association between anxiety and depression scores and DHM indicators (volume received, age of the baby at the end of support). The only significant associations were found between depression scores and the volume of milk received. A significant negative association was found between the volume of milk received and post‐depression score (*r* = −0.341, *p* = 0.002). The larger the volume of milk received, the lower the depression score. A larger volume received was also significantly associated with a greater drop in depression score (*r* = 3.16, *p* = 0.004). No significant association was seen with predepression score (*r* = 0.086, *p* = 0.448).

A series of *t*‐tests examined differences in anxiety and depression scores between those who received exclusive or partial DHM. No significant differences were found in pre [*t* (78) = 0.852, *p* = 0.198], post [*t* (78) = −0.127, *p* = 0.449] or change [*t* (78) = 0.719, *p* = 0.237] anxiety scores. Similarly, no significant differences were found in pre [*t* (78) = 1.22, *p* = 0.112], post [*t* (78) = −0.102, *p* = 0.155], or change [*t* (78) = 1.52, *p* = 0.065] depression scores.

As described in the methods, reasons for seeking donor milk were grouped into two categories: maternal health and low supply. Overall, 39 (48.8%) were classed as maternal health reasons and 41 (51.2%) as low supply. Those in the maternal health group received a significantly higher volume of milk than those in the low supply group [*t* (78) = 4.325, *p* < 0.01]. An average of 25.32 L (SD: 12.06) were received by those in the health group and 13.73 (SD: 11.91) in the low supply group. A chi‐square test also found a significant association between reason and exclusive/partial use [*χ* ^2^ = 38.23, *p* < 0.001]. Overall, 64.1% of those in the maternal health group received exclusive supply while all in the low supply group received a partial supply.

A series of *t*‐tests found a significantly higher rate of pre‐DHM depression in the maternal health issue compared to the milk supply issue group [*t* (78) = −2.578, *p* = 0.006]. No significant difference was found in post‐DHM depression scores [*t* (78) = 0.952, *p* = 0.172] with a significant difference in pre‐ and post‐DHM depression score difference [*t* (78) = −2.263, *p* = 0.013]. Those in the maternal health group decreased by an average of 4.67 (SD: 3.37) points compared to an average decrease of 2.97 (SD: 3.20) in the milk supply group.

For lactation support, the mean number of emails sent was 8.29 (SD: 7.10 with a range from 0 to 38) and the mean number of calls made was 8.84 (SD: 4.21 with a range from 2 to 24). Combining both emails and calls together, the mean number of contacts was 17.12 (SD: 9.42 with a range from 2 to 50). No significant associations were found between preanxiety (*r* = −0.042, *p* = 0.709), post‐anxiety (*r* = 0.165, *p* = 0.142), pre‐depression (*r* = 0.173, *p* = 0.125) or post‐depression (*r* = −0.109, *p* = 0.092) scores and number of contacts (email and calls combined). A significant association was however found between number of contacts and change in depression score. The more contacts a parent received, the greater their reduction in depression score (*r* = 0.250, *p* = 0.026). A higher volume of milk was significantly related to a greater amount of support (*r* = 0.414, *p* ≤ 0.01), but no significant difference in the degree of support was found between partial and exclusive use [*t* (78) = −0.044, *p* = 0.482].

Three factors were therefore associated with a difference in depression score: volume of milk, number of support contacts and reason for seeking milk. In a stepwise regression analysis [*F* (1, 78) = 8.350, *p* = 0.005, *R* ^2^ = 0.85], only volume of milk remained a significant predictor (*p* = 0.005).

### 3.1 Thematic analysis

A thematic analysis was conducted to explore experiences of receiving DHM upon mental health and wellbeing. All participants responded to at least some of the open‐ended questions. Examples of quotes are given below, with details of the participant: parent, age, exclusive/partial use of DHM, and maternal health or low supply issue (specific details not given to avoid potential identification). Participants often described broad impacts upon how receiving DHM had impacted their mental health, going on to elaborate on how this impact occurred. Overall, the strength and depth of the impact of receiving DHM came through clearly in their responses:

There is little doubt in my mind that without the donor milk I would have sunk into deep depression/anxiety, as I found it very distressing to give my baby formula – it made me feel like a failure and a bad mother, and to have big worries about her future health, and was impacting our bonding process. This would have persisted well into her childhood. As a result, donor milk has meant the world to me and has allowed me space to enjoy motherhood and bond with my baby, and to ‘reframe’ myself as a supermum who went above and beyond for her little one. The relief of knowing that she has received good nutrition is difficult to express. (Mother, 32, partial, low supply)

Examining the ways in which participants elaborated on how receiving milk impacted upon their health and wellbeing, six themes were identified.

#### 3.1.1 Theme one: Reducing anxiety around infant health

Many parents in the study described how, first and foremost, receiving DHM helped improve their wellbeing because it reduced anxiety over their baby's current or future health. For some parents, this concern was not related to a specific (or current) health issue but rather centred on knowing how human milk protects infant health or worrying about the impacts of using formula milk.

My baby has had a very sensitive gut and a potential dairy allergy, so it's a relief to know that he got those first few weeks of exclusive breastmilk rather than going straight onto a dairy based formula. (Mother, 32, exclusive, maternal health)

Receiving the donor milk gave me reassurance that my son was being fed in the best way possible which was an enormous weight off my mind during a very stressful time. He was quite small when he was born but after feeding on the donor milk he jumped up a centile line within 3 weeks – it's wonderful! (Mother, 40, exclusive, maternal health)

Our baby had four weeks of donor milk which we think gave her a better start than formula alone. (Father, 40, partial, low supply)

Other parents specifically raised health issues in relation to complex health issues. DHM played a particular role in helping ease anxiety around vulnerable infants.

I have been diagnosed with IGT so I cannot make enough. I make around half his feeds. Some days more. In terms of the difference to me, my son was prem. he also had emergency surgery. It helped him so much. Also for my own mental health, my 3 year old son is cognitively disabled he cannot talk due to an unknown development issue. (Mother, 29, exclusive, maternal health)

Because of this milk it has meant that my son hasn't had to go into hospital to be fed via an ng tube. (Mother, 25, partial, low supply)

#### 3.1.2 Theme two: Taking the pressure off

Receiving DHM often took the pressure off mothers who were trying to build their own supply or to express enough for an unwell baby who could not feed directly.

It was amazing. It was so helpful and took lots of pressure off of myself while going from breastfeeding to exclusively pumping. My baby has only been fed human milk and it was really important to me. (Mother, 27, partial, low supply)

It was a relief to know my baby was not going to be hungry anymore. I felt very sad I couldn't breastfeed enough as my milk supply was very low but while I tried everything to increase it the donor milk helped me and my baby. I was suddenly much less stressed. (Mother, 36, partial, low supply)

#### 3.1.3 Theme three: Privilege and gratitude

Privilege was a word often used, especially by mothers in the sample. Many realised that this opportunity was not widely scaled up across the United Kingdom and felt lucky to be able to be offered the option. These feelings often helped to ease the very challenging experiences that families found themselves in.

I felt so grateful to have this opportunity, especially when I realised many do not. It gave me time to work on building my own supply, a respite for which I will always be grateful. (Mother, 36, partial, low supply)

It's one of the most valuable and meaningful experiences of my life. In a time of real crisis, having been diagnosed with breast cancer, words can only scratch the surface of what it means to me to have received this milk for my baby. I cannot be more grateful. (Mother, 39, exclusive, maternal health)

Alongside this, women often described the impact of knowing other women had taken the time to donate their milk. This kindness and generosity helped them to feel supported and as part of a bigger circle of other mothers feeding their babies.

It hadn't occurred to me until sitting feeding my baby in the small hours of the morning, that donor breastmilk would also carry with it, for me at least, a strong sense of solidarity and support from the other mothers who donated that milk. I still find that incredibly moving and consoling. (Mother, 41, partial, low supply)

#### 3.1.4 Theme four: Emotional support

Many mothers recognised the role that emotional support played in boosting their wellbeing during the process of requesting and using DHM.

It was amazing to have the support for as long as we did & we were so very grateful. It was incredible to have that emotional support from the team too and to know that my baby is currently EBF (if not by me) is so important to me and my mental health too. (Mother, 35, partial, low supply)

A core part of this was feeling listened to and respected. It was common for mothers to feel that others did not understand why they felt distressed at not being able to breastfeed (or do so fully) or their desire to use DHM. Feeling heard and having their experience validated helped support their mental health separately to being able to receive DHM.

Before contacting [the service], I was made to feel that even though we have a problem and I can't feed my baby the way I would like to, my problem is lesser, and thus doesn't need solving. In general, I found Insufficient Glandular Tissue to be completely ignored as an issue – the midwives dismissed my worries and just told me to feed my baby formula. I was depressed and worried that I wasn't providing anything good for my baby. [The service] was the first place that took me seriously and instantly provided some help. Being able to get my son more breastmilk, with the knowledge that my body couldn't produce enough, was absolutely priceless. (Mother, 33, partial, maternal health)

#### 3.1.5 Theme five: Healing from the experience of not being able to breastfeed

Related to the previous theme was the importance of feeding preferences being respected and supported. Receiving DHM helped some women to heal from the experience of not being able to breastfeed themselves or do so exclusively.

I was heartbroken when I was unable to provide breastmilk for my son. Being given donor milk helped to heal that somewhat, as I knew he was getting most of the benefits of breastmilk in those first crucial weeks of his life. I guess I felt a bit less like I'd failed him. It's hard to say what difference it made to the baby, but he certainly enjoyed it and seemed to digest it well. (Mother, 37, partial, maternal health)

Our sample included women with complex health issues that affected their ability to breastfeed. It was notable that the experience of receiving DHM was so important for these mothers that it also helped them to process the emotional impact of their health diagnosis.

It meant the world to me to know that my daughter was able to receive human milk as prior to my breast cancer diagnosis, I always intended to breastfeed for over a year. It was extremely difficult to come to terms with having to stop. It was harder to accept than my cancer diagnosis. (Mother, 39, partial, maternal health)

#### 3.1.6 Theme six: Difficult emotions

However, not all emotions experienced were positive. Feelings of failure at not being able to breastfeed or experiences around using DHM sometimes went alongside the positives.

It upset my partner that we had to use somebody else's milk, but we were happy to be able to feed our baby. (Father, 38, partial, low supply)

At first it was difficult to accept that we could not exclusively breastfeed. My partner disagreed on the amount of donor milk we should be using. He wanted to use more because he wanted to make sure our baby was well fed, I was worried about how the donor milk would impact my own supply. (Mother, 39, partial, low supply)

#### 3.1.7 Theme seven: Impact of receiving DHM upon breastfeeding

Some mothers in the study were unable to give their babies their own breast milk due to health issues such as cancer treatment. Others, however, received DHM alongside their own breast milk. Often, these mothers were working on increasing a low milk supply, and many of those within this category commented about the impact DHM had upon this experience. Although these mothers were not directly asked about how DHM supported breastfeeding, it was often mentioned in the comments.

As noted in theme two, part of the positive impact of receiving DHM was the reduction in immediate pressure to provide milk, which in turn reduced anxiety. It also appeared to help mothers build their own supply and continue feeding. Part of this was physiological. The reduced pressure helped with relaxation, which in turn helped a mother produce more milk. Supply often rose given time, and this reduction in anxiety.

It was lifesaving, there are no words to express the gratitude and explain the relief I felt. A huge weight was lifted from my shoulders and I felt I could finally breathe and feed my baby. I did everything for my milk supply (fenugreek, linseed, porridge, yeast, domperidone, lots of water, rented hospital grade pump) and it finally came and now my baby is back on her percentile, happy and beautiful. (Mother, 36, partial, low supply)

However, the relationship was deeper than this and included aspects such as increasing determination to provide more milk themselves, wanting an exclusive human milk diet for their baby, or feelings of gratitude at what other women had done through donating spurring them on.

I am now pumping more than my boy needs so it's helping me feel secure in ensuring he received only breastmilk for as long as possible. (Mother, 27, partial, low supply)

I felt I owed it to those who donated to do everything I could and I'm certain this helped us build our supply to where we are now. (Mother, 34, partial, low supply)

## 4 DISCUSSION

Using a pre‐ and post‐intervention design, this study examined the impact of receiving DHM upon parental mental health. Our data showed a significant decrease in both anxiety and depression as measured by the HADS after receiving DHM, with a significant decrease in the number of parents categorised as having borderline or abnormal anxiety and depression scores. Before receiving DHM, 7.5% of the sample had ‘normal’ levels of anxiety, increasing to 48.8% post‐receiving milk. Likewise, for depression, 3.8% had normal levels of anxiety before receiving milk, increasing to 57.5% post‐receiving milk. Meanwhile, the average change of 7.5 points on the HADS combined scale exceeds what would be considered ‘meaningful’ in other areas of health research (Lemay et al., 2019; Longo et al., 2023; Puhan et al., 2008). Our work builds on qualitative work on this topic that suggests a protective impact of receiving DHM upon parental (predominantly maternal) wellbeing (Brown & Shenker, 2022; Cassidy & Dykes, 2019; Kair & Flaherman, 2017; McCloskey & Karandikar, 2019) but is the first to quantitatively measure symptoms of anxiety and depression and changes over time. Although there are limitations to the work, our findings add an important consideration to the evidence around the benefits of access to DHM for both infants and their parents.

The relationship between infant feeding and wellbeing is complex. We know that a short breastfeeding duration, or experiencing breastfeeding difficulties and pain can negatively affect maternal wellbeing, particularly if infants are unwell (Avilla et al., 2020; Brown, 2018; Flacking et al., 2016; Hookway et al., 2023; Shepherd et al., 2017). However, little research has considered how DHM may play a role in this, perhaps previously considering DHM as a medical intervention or something that infants receive from a medical team rather than part of infant feeding and relationship building. Our findings show that DHM should be considered in studies that examine infant feeding and wellbeing, especially as DHM is often given alongside MOM, or reduces or prevents the introduction of formula milk.

Our findings also reinforce the need for feeding and lactation support to be given alongside access to DHM. When mothers want to breastfeed but are unable to do so, part of their distress is related to their infant and potential health impacts (Lagan et al., 2014), but mothers are also affected by a lack of support or understanding of the importance of breastfeeding to them due to cultural, social, health, or personal considerations (Brown, 2019; Hvatum & Glavin, 2017; Penniston et al., 2021). It is likely that for mothers for whom human milk feeding is important, receiving DHM also reflects those values. Our findings have clear implications for those working to support families with DHM but also for infant feeding more broadly. Parents did talk about the reassurance of receiving DHM for their infant's health, but also highlighted how their experiences of feeling that their infant feeding choices were respected and supported and receiving care and reassurance helped to support their wellbeing.

However, when discussing wellbeing, attention should be drawn to the small number of mothers who described feelings of guilt or distress at not being able to breastfeed and having to use another woman's milk. This reflects a small theme in qualitative research exploring the impact of receiving donor milk (Brown & Shenker, 2022), which also introduced the idea of feeling guilty that their baby was able to access DHM when other babies might not be able to. Guilt can be a complex emotion around infant feeding. Women can feel guilt when unable to breastfeed their baby (Jackson et al., 2021), but also guilt for receiving donated milk from another mother or instead of it going to another baby. This was also reflected in a study with Australian mothers who had a baby in neonatal care and considered using donor breast milk. Some felt that it exacerbated their feelings of failure and that other mothers were doing better than them by having such a good supply of milk that they could also support other babies (Zizzo, 2013).

It should be noted that in the milk bank included in this study, donor milk for ‘community participants’ whose babies are full term and healthy but their mother needs additional milk typically comes from donors who may not meet the stringent donation standards needed for donor milk prepared for more vulnerable babies in the neonatal unit. These donors typically are breastfeeding beyond 6 months post‐natally or are taking a medication deemed by a specialist pharmacist to be safe for donation to full‐term, healthy infants. DHM is not diverted from neonatal units. This is an important aspect of ensuring parents whose baby receives this milk understand that, although other families might not benefit currently from receiving it, it is not in place of a baby in neonatal care being able to access it.

A key question we had was whether any aspect of the experience, that is, volume, exclusivity reason or support received, were associated with anxiety and depression scores. This insight may help with service planning and consideration of who may benefit the most from DHM and in which way. No aspect of receiving milk was significantly associated with pre, post or change in anxiety score. Potentially, simply being part of the programme and receiving the DHM and support alongside it helps to reduce maternal anxiety with no significant variation based on experience.

However, some significant factors arose for depression. Mothers who received DHM for a maternal health issue (such as bilateral mastectomy or developmental anomaly) had significantly higher pre‐depression scores (and subsequent reduction) than those who sought it due to a milk supply issue (such as partial supply, surrogacy or infant health). It is possible that this is due to maternal health issues such as cancer bringing greater stress; however, seeking milk for an older, unwell infant would likely also indicate significant stress. Mothers who were seeking milk for their own health reasons typically could not breastfeed at all, whereas women in the milk supply group either had a partial supply or were seeking milk in circumstances where they might not be expected to be producing a supply (i.e., for an older infant). Potentially, mothers in the health issue group hold greater feelings of guilt, grief or anger at not being able to breastfeed at all, exacerbated by health issues.

We also considered access to feeding and lactation support. The more contacts a parent received, the greater their reduction in depression symptoms. However, again, when the volume of milk was controlled for, this relationship disappeared. Potentially, parents who received more milk naturally received more support. The lactation support may have enabled donor milk to have been received for longer as it ensured that milk was used correctly, and in a way that supported increasing milk supply if that was the goal. This does not mean that feeding and lactation support are not important, and mothers, in particular those who were breastfeeding, often described their importance in open‐ended comments. However, instead, perhaps all parents in the study had sufficient support. All received one‐to‐one individualised support, which was tailored to their needs, that is, some parents needed greater support than others, and some parents gravitated to phone rather than email support. We know that receiving tailored, regular support with breastfeeding helps to increase the duration (McFadden et al., 2019) and improve maternal mental health (Pezley et al., 2022). This also raises questions around how parents are supported if DHM is unavailable. What role might therapeutic support play in helping reduce negative mental health outcomes linked to infant feeding experiences?

However, it was only the volume of milk received that was significantly associated with a drop in depression score and remained predictive in the regression analysis. Our sample is too small to reliably consider whether there could be a standardised ‘dose’ of volume of DHM that might help to protect mental health, and even in a larger sample, this is likely to be affected by many experiential and contextual factors. It could simply be that receiving more DHM provides significantly greater reassurance and a feeling of being listened to and supported. Alternatively, a greater desire or need for milk (and thus a greater potential reduction in score) may affect the volume given. Provision of DHM by the charity is not standardised in volume. Consideration is given to the long‐term nutritional needs of an infant and whether these will be fully met by pasteurised DHM, as well as to the overall demand. Parents work closely with a lactation support lead with the volume provided dependent on a combination of need, age and availability of milk. It is possible that parents who place the greatest value on human milk or who show the greatest distress are given or request more milk (although the level of support was unrelated to pre‐anxiety and ‐depression scores). Further research is needed to explore parents' perception of how the volume of milk affects their experience, with consideration of how support needs to be personalised to families and how the quality of personalised care can be maintained as demands for milk bank services expand.

An important consideration is whether anxiety and depression may naturally reduce over time and whether or not DHM is received. At the start of the study, parents would likely have been under considerable stress due to events surrounding the birth, maternal health issues, and needing to source DHM. These may have eased over the duration of the study, and we made the decision not to have a comparator group who did not receive DHM due to the potential stress of asking parents who had not been able to access DHM (after requesting but none being available or lactation support being more appropriate) to participate. However, we found no significant association between changes in anxiety and depression scores and the duration or milk received or the age of the baby at the end of DHM. Parents who completed it after a short duration of DHM, whose baby was typically younger, had no difference in anxiety or depression scores compared to parents of older infants. Findings from research examining the longitudinal prevalence of post‐natal depression after birth are also mixed. Some studies find that prevalence remains similar (Rubertsson et al., 2005) or new cases emerging over time (Kikuchi et al., 2021) from post‐birth to 1 year, whereas others suggest a decrease (Gavin et al., 2005) or variance between women depending on other contextual factors (Sutter‐Dallay et al., 2012). However, importantly, when asked, almost all participants in our study attributed being able to access DHM and the supportive process of receiving it helped to improve their mental health and wellbeing.

Two further questions are whether these findings would differ if conducted in a neonatal sample and how parental perceived value of DHM affects the outcome. Our sample represents parents who were in challenging circumstances, often around the health of themselves or their baby, and who were highly motivated and aware of DHM to seek it out. They are unusual within a post‐natal context where most of their peers who needed to supplement likely used formula milk. In a neonatal context, at least for the most premature infants, DHM is more likely to be offered and seen as a ‘usual’ option. Does this availability affect the impact on parents when received? Further research is needed to understand the potential scale of the impact of DHM upon parental health and its implications on national and local milk banking strategies and future planning.

Although the focus of this study was examining the impact of receiving DHM upon mental health, the data show the potential impact of DHM upon mothers building their own supply and continuing to breastfeed. Almost all mothers who received a partial supply of DHM continued to also provide maternal milk, with those who were not unable to do so due to health reasons. It was notable that 84% of those babies who received maternal milk continued to be breastfed after DHM was completed, 54.8% of them exclusively. In the open‐ended qualitative comments, mothers described how receiving DHM helped to support them to maximise their own milk supply where possible. When DHM was used alongside MOM as a supplement, mothers reflected that it helped motivate them to continue building their own supply to be able to avoid formula supplementation or to continue giving their baby human milk longer term.

This may partly be due to a very motivated sample who valued human milk, but it also shows the importance of ensuring that mothers who can provide maternal milk are supported to continue to do so once DHM is introduced. As one mother in the sample noted in the open‐ended boxes, there was a mismatch between her partners' view (give as much DHM as possible) and her own desire to limit it to ensure her own supply continued to build. Awareness of this potential conflict or risk of maternal own supply decreasing must be considered. Our study showed that all mothers also giving maternal milk received lactation support from an IBCLC, emphasising this as an important part of DHM provision.

This adds to growing evidence that DHM may play an important role in supporting mothers to increase their own supply where possible, as DHM acts as an exclusive human milk diet ‘bridge’ to full breastfeeding. DHM has been associated with increased rates of breastfeeding at hospital discharge (Williams et al., 2016) and a five‐fold increase in exclusive breast milk feeding at 6 months (Merjaneh et al., 2020). Qualitative data has also highlighted how mothers attribute receiving DHM to supporting their motivation to continue providing exclusive human milk for their infant, in part due to the reasons above but also because they felt positively indebted to those who had donated milk and wished to honour them by continuing (Brown & Shenker, 2022; Kair & Flaherman, 2017). Further research is needed as to the impacts of using DHM as a bridge to support breastfeeding on neonatal, post‐natal and paediatric wards.

Another aspect to consider in further research is the impact upon infant health that providing DHM to families outside of the typical NICU system might bring. DHM may help to reduce the occurrence and severity of necrotising enterocolitis and complications such as bronchopulmonary dysplasia in premature infants (Quigley et al., 2019; Villamor‐Martínez et al., 2018). Significant evidence exists that MOM protects infant health and development in term infants (Victora et al., 2016). We do not however know the impact upon infant health of DHM for term and older infants. Parents in our sample described how they felt that DHM helped support their baby's health (and in some cases, prevent hospital admission) due to a history of allergy in the family, emergency surgery, or slow weight gain. Although pasteurisation does reduce the immunological properties of breast milk (Rodríguez‐Camejo et al., 2020), infant formula does not contain these properties. In the absence of MOM, DHM may, therefore, likely offer protection to term and older infants.

Our study does have limitations. The sample size was relatively small, although it extends the size of most previous research in this area. Our participants were from only one milk bank due to wanting to focus on parents who received DHM outside of the NICU setting (where more research has been conducted). The milk bank studied had a large community donor milk programme and although DHM has been provided by NHS led milk banks this is typically less frequently. The unique arrangement of requesting milk with typically a short delay until provision enabled the pre‐ and post‐design to be delivered. The study could be completed within neonatal care units, but typically, DHM is offered as standard to infants who meet criteria (usually <32 weeks gestation), with reduced time for pre‐test completion.

As with any screening tool, the HADS has limitations and may underestimate depression in those scoring below the cut‐off. It has also been criticised for not measuring fatigue and sleep disturbance, although given high levels of these in post‐natal parents, this may not apply to our study (Moulton et al., 2019). We grouped reasons for receiving DHM into two main categories (a supply issue and a health issue) to create two larger groups for broad comparison but realise this may have limitations and reasons for seeking DHM. A larger sample would allow comparative analysis by specific reasons rather than grouping them together.

Our participants were also older than average with a higher level of education and were self‐selecting, both in terms of sourcing DHM in the first place and choosing to take part in the research. Although all parents who received DHM were invited to take part in the research, not every parent did. It is possible that those with the greatest desire to receive DHM or the most positive experiences chose to take part in the research. However, this is an issue with all survey research and our demographics reflect other hospital‐based research on this topic but may also highlight disparities in who is aware of DHM and potential opportunities to access it outside the neonatal care unit. Our pre‐completion rate was also higher than our post‐completion rate, but this is likely because completing a longer survey in the post‐natal period can be challenging.

In addition, geographically, our sample was based predominantly within a single region of England, although some distance and variation was found between families. All participants were attached to one community milk bank, but given the specific nature of receiving milk (e.g., for maternal health or low supply rather than infant prematurity), it is unlikely that collecting data from parents at other hospital‐based milk banks would have expanded our sample as they primarily serve neonatal units.

We decided to use a survey because it allowed participant anonymity when describing what might be difficult emotions and experiences. It also enabled participants to take part quickly and easily at a time of their choosing, which was especially important to us due to the added demands of caring for an infant combined with maternal health or feeding complications. Additionally, the limited research in this area tends to be qualitative in design. Our mixed methods survey, which included open‐ended boxes, allowed both quantitative data using an established tool and qualitative responses to be collected. However, further work may wish to explore the mental health impacts of receiving donor milk through in‐depth interviews.

We also chose to include fathers/partners in our study because they are often instrumental in arranging DHM provision or maybe caring solely for their infant in cases of adoption or maternal ill health or bereavement. Our sample size is not large enough to examine statistical mental health impacts, but a reduction in anxiety and depression scores was seen for all fathers in our study, at a similar rate to mothers in our study. This supports findings from a previous study where fathers described how being able to source DHM for their infant helped support their wellbeing (Brown & Shenker, 2022). We also know that supporting breastfeeding and breast milk can be important to fathers (Sihota et al., 2019), and they can carry a feeling of responsibility for caring for their partner and new baby (Rempel et al., 2017). Fathers/partners may not carry the same complexity of emotions if their baby is not breastfed but may likely be affected by broader anxieties around their infant, their partners' wellbeing and, in our study, often maternal health. Further research into fathers/partners and infant feeding experiences is warranted, potentially as a stand‐alone study or with targeted recruitment.

## 5 CONCLUSION

To conclude, our data add an important aspect to the discussion around infant feeding and mental health, highlighting for the first time using quantitative data that receiving DHM may help to protect parental mental health at a challenging time, significantly reducing anxiety and depression scores. Further work is needed to validate the findings in a larger and broader sample, extending inclusion to those who have not sought out such a service. This could include participants who had received DHM for a baby in neonatal care and, therefore, include a broader range of settings from across the United Kingdom. Data is also needed to understand the potential costs‐savings to the NHS through reduced infant morbidity, re‐admissions and improved maternal mental health in particular. The role of how such a community‐facing lactation support service works and what resources and infrastructure are needed to upscale this should be considered.

## AUTHOR CONTRIBUTIONS

Amy Brown was responsible for study conception, study design, data collection, data analysis and draft report writing. Natalie Shenker was responsible for study conception and coordination, data collection and draft report writing. Sam Griffin coordinated data collection. Gillian Weaver was responsible for study conception, data interpretation and draft report writing. All authors read and approved the final manuscript.

## CONFLICT OF INTEREST STATEMENT

The authors declare no conflict of interest.

## Supporting information

Supporting information.

## Data Availability

Data are available on request.
