# Supplementary material for: Receiving screened donor human milk as part of a community‐based lactation support programme reduces parental symptoms of anxiety and depression
Source: Matern Child Nutr. 2024 Jun 19;20(4):e13686. doi: 10.1111/mcn.13686 (PMC11574670; doi:10.1111/mcn.13686)
Supplement: Supplementary file 1 — Supporting information. [file MCN-20-e13686-s001.docx]

**Pre receiving milk survey items**

Q1 Recipient number

|  |
| --- |

Q2 What is your sex?

|  |
| --- |

Q3 How old are you?

|  |
| --- |

Q4 What is your highest level of education?

- No formal qualifications (1)
- GCSE or equivalent (2)
- A level or equivalent (3)
- Degree or equivalent (4)
- Postgraduate degree or equivalent (5)

Q5 What is your marital status?

- Married / civil partnership (1)
- Cohabiting (2)
- Single (3)
- Divorced (4)
- Widowed (5)

Q6 Are you currently employed?

- Yes full time (10)
- Yes part time (11)
- No (12)

Q7 What is your ethnicity?

- White / White British (1)
- Gypsy / Traveller (2)
- Irish (3)
- Asian or Asian British: Pakistani (4)
- Asian or Asian British: Bangladeshi (5)
- Asian or Asian British Indian (6)
- Asian or Asian British: Chinese (7)
- Asian or Asian British: Other (8)
- Black or Black British (9)
- Mixed or multiple (10)
- Other (11)
- Prefer not to say (12)

Q8 How many children do you have?

|  |
| --- |

Q9 How many babies / children are you seeking donor milk for?

|  |
| --- |

Q10 If they have been born, are they male or female?

|  |
| --- |

Q11 If they have been born, how old are they?

|  |
| --- |

Q12 If your baby has been born, how are they currently fed? Please complete for each baby you are seeking donor milk for i.e. if twins or triplets etc

|  | Baby one (1) | Baby two (2) | Baby three (3) |
| --- | --- | --- | --- |
| Only breastmilk from birth (1) |  |  |  |
| Now only breastmilk but had formula milk in the past (2) |  |  |  |
| A mix of breast and formula milk (3) |  |  |  |
| Only formula milk (4) |  |  |  |

Q13 Have you previously received any donor milk? Please tick all that apply

- Yes in hospital (1)
- Yes from a milk bank when we were home from hospital (2)
- Yes another mother shared their milk with me (3)

Q14 I hope that receiving donor milk will help:

|  | Strongly agree (1) | Somewhat agree (2) | Neither agree nor disagree (3) | Somewhat disagree (4) | Strongly disagree (5) |
| --- | --- | --- | --- | --- | --- |
| My baby’s health (1) |  |  |  |  |  |
| My baby’s development (2) |  |  |  |  |  |
| My mental health (3) |  |  |  |  |  |
| My physical health (4) |  |  |  |  |  |
| My family’s wellbeing (5) |  |  |  |  |  |

Q15 Reading through the next statements, please choose the one that fits most closely with how you have felt in the past week?

|  | Most of the time (1) | A lot of the time (2) | Occasionally (3) | Never (4) |
| --- | --- | --- | --- | --- |
| I feel tense or wound up (1) |  |  |  |  |
| I still enjoy the things I used to enjoy (2) |  |  |  |  |
| I feel cheerful (3) |  |  |  |  |
| I feel as if I am slowed down (4) |  |  |  |  |
| I get a sort of frightened feeling like ‘butterflies’ in the stomach (5) |  |  |  |  |
| I get sudden feelings of panic (6) |  |  |  |  |
| I can enjoy a good book or radio or TV program (7) |  |  |  |  |
| I feel restless as I have to be on the move (8) |  |  |  |  |
| I can sit at ease and feel relaxed (9) |  |  |  |  |

Q16 I get a sort of frightened feeling as if something awful is about to happen

- Definitely and quite badly (1)
- Yes, but not too badly (2)
- A little, but it doesn’t worry me (3)
- Not at all (4)

Q17 I can laugh and see the funny side of things:

- As much as I always could (1)
- Not quite so much now (2)
- Definitely not so much now (3)
- Not at all (4)

Q18 Worrying thoughts go through my mind

- A great deal of the time (1)
- A lot of the time (2)
- From time to time, but not too often (3)
- Not at all (4)

Q19 I have lost interest in my appearance

- Definitely (1)
- I don’t take as much care as I should (2)
- I may not take quiet as much care (3)
- I take just as much care as ever (4)

Q20 I look forward with enjoyment to things

- As much as I ever did (1)
- Rather less than I used to (2)
- Definitely less than I used to (3)
- Hardly at all (4)

**Post receiving milk survey items**

Q4 Recipient number

|  |
| --- |

Q13 How old was your baby / babies when they first received donor milk?

|  |
| --- |

Q40 What was your experience of receiving donor milk? For example, how did it make you feel or what difference did it have to you, your baby or your family?

|  |
| --- |

Q47 How did your experience of donor milk fit with your expectations of receiving donor milk? Was it better? Different? More challenging than expected?

|  |
| --- |

Q18 How are you feeding your baby / babies now? Please tick all that apply

|  | Baby one (1) | Baby two (2) | Baby three (3) |
| --- | --- | --- | --- |
| My own breast milk (1) |  |  |  |
| Donor milk from a milk bank (2) |  |  |  |
| Donated milk shared by another mother (3) |  |  |  |
| Formula milk (4) |  |  |  |

Q42 If you were breastfeeding your baby but are no longer doing so, how old were they when you stopped?

|  |
| --- |

Q44 If you were giving your baby formula milk, but are no longer doing so, how old were they when you stopped?

|  |
| --- |

Q45 How happy are you with how you are currently feeding your baby?

- Extremely happy (1)
- Somewhat happy (2)
- Neither happy nor unhappy (3)
- Somewhat unhappy (4)
- Extremely unhappy (5)

Q46 How do you feel about how you are currently feeding your baby? Can you tell us a little more about why you feel that way?

|  |
| --- |

Q27 Reading through the next statements, please choose the one that fits most closely with how you have felt in the past week?

|  | Most of the time (1) | A lot of the time (2) | Occasionally (3) | Never (4) |
| --- | --- | --- | --- | --- |
| I feel tense or wound up (1) |  |  |  |  |
| I still enjoy the things I used to enjoy (2) |  |  |  |  |
| I feel cheerful (3) |  |  |  |  |
| I feel as if I am slowed down (4) |  |  |  |  |
| I get a sort of frightened feeling like ‘butterflies’ in the stomach (5) |  |  |  |  |
| I get sudden feelings of panic (6) |  |  |  |  |
| I can enjoy a good book or radio or TV program (7) |  |  |  |  |
| I feel restless as I have to be on the move (8) |  |  |  |  |
| I can sit at ease and feel relaxed (9) |  |  |  |  |

Q28 I get a sort of frightened feeling as if something awful is about to happen

- Definitely and quite badly (1)
- Yes, but not too badly (2)
- A little, but it doesn’t worry me (3)
- Not at all (4)

Q29 I can laugh and see the funny side of things:

- As much as I always could (1)
- Not quite so much now (2)
- Definitely not so much now (3)
- Not at all (4)

Q30 Worrying thoughts go through my mind

- A great deal of the time (1)
- A lot of the time (2)
- From time to time, but not too often (3)
- Not at all (4)

Q31 I have lost interest in my appearance

- Definitely (1)
- I don’t take as much care as I should (2)
- I may not take quiet as much care (3)
- I take just as much care as ever (4)

Q32 I look forward with enjoyment to things

- As much as I ever did (1)
- Rather less than I used to (2)
- Definitely less than I used to (3)
- Hardly at all (4)

Q33 Finally, do you have any further comments on the experience of receiving donor milk?

|  |
| --- |
